# Corrigendum to: A quantitative systems pharmacology approach to support mRNA vaccine development and optimization

**DOI:** 10.1002/psp4.12767

**Published:** 2022-03-03

Gianluca Selvaggio, Lorena Leonardelli, Giuseppe Lofano, Stephanie Fresnay, Silvia Parolo, Duccio Medini, Emilio Siena and Luca Marchetti, *CPT Pharmacometrics Syst Pharmacol*. 2021;10:1448–1451, https://doi.org/10.1002/psp4.12721.

In the published version of Selvaggio G, et al. (2021)^1^ the fifth author’s name was misspelled as Silivia Parolo and should instead be Silvia Parolo.

The authors apologize for the error.
